# Supplementary figures and images for: Characteristics and expression profiles of circRNAs during abdominal adipose tissue development in Chinese Gushi chickens
Source: PLoS One. 2021 Apr 15;16(4):e0249288. doi: 10.1371/journal.pone.0249288 (PMC8049301; doi:10.1371/journal.pone.0249288)

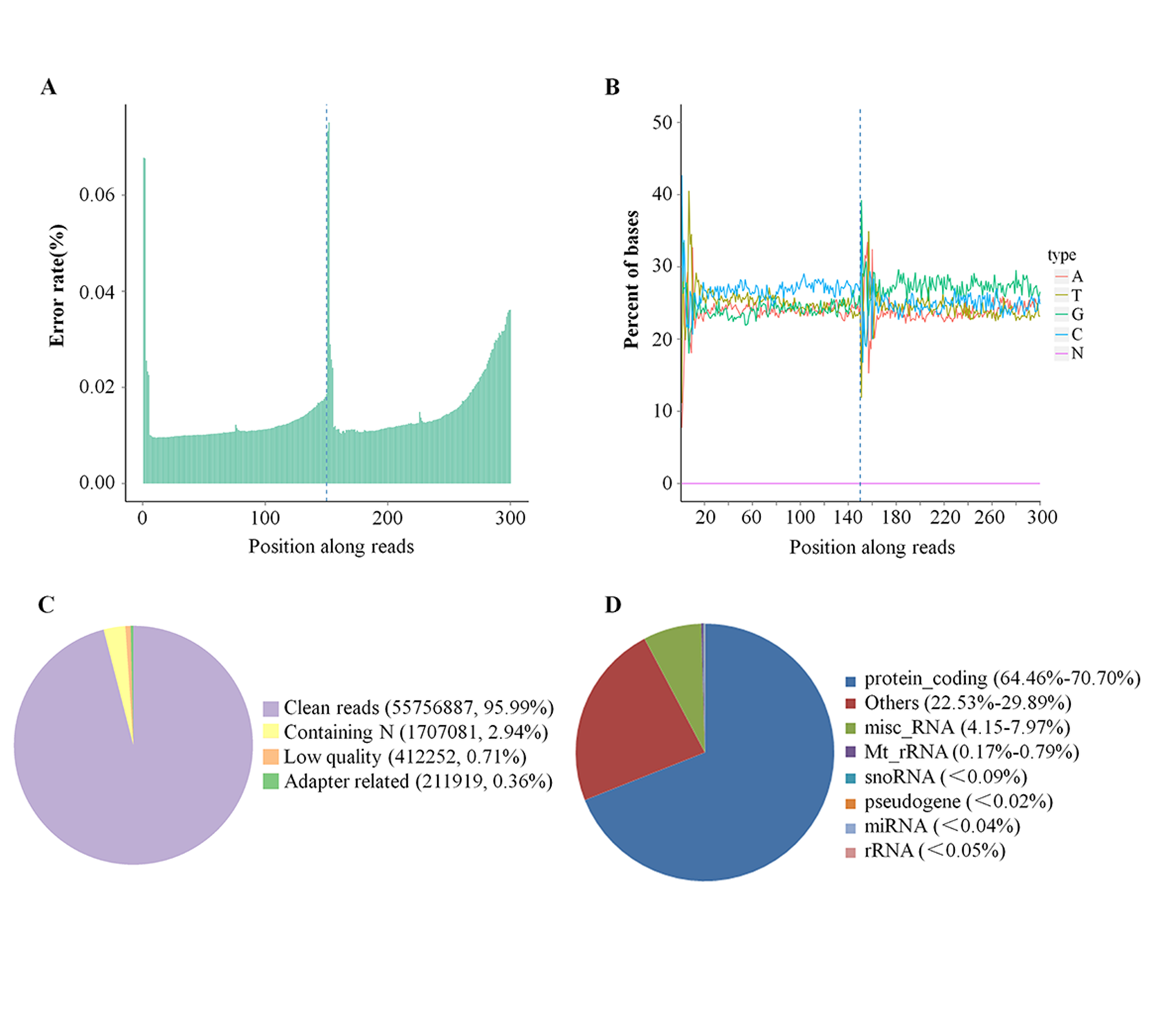

Supplement: S1 Fig — (A) The sequencing error rate of each base position. (B) The content of each base during the sequencing process. (C) The proportion of different types of raw data. (D) Classification of mapped reads in 12 libraries. (TIF) [file pone.0249288.s001.tif]

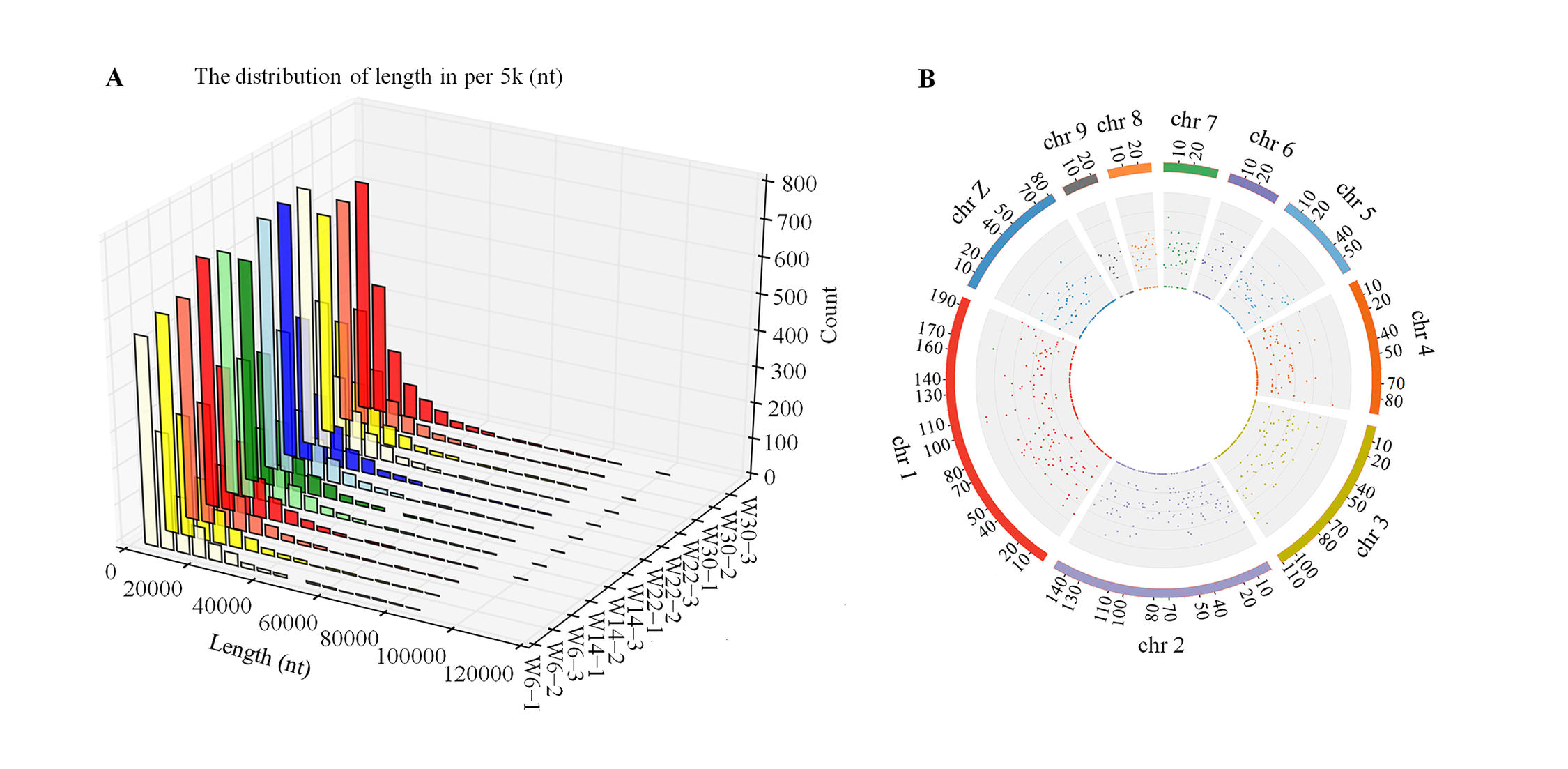

Supplement: S2 Fig — (A) Length distribution. (B) The distribution of circRNAs in different chromosomes. (TIF) [file pone.0249288.s002.tif]

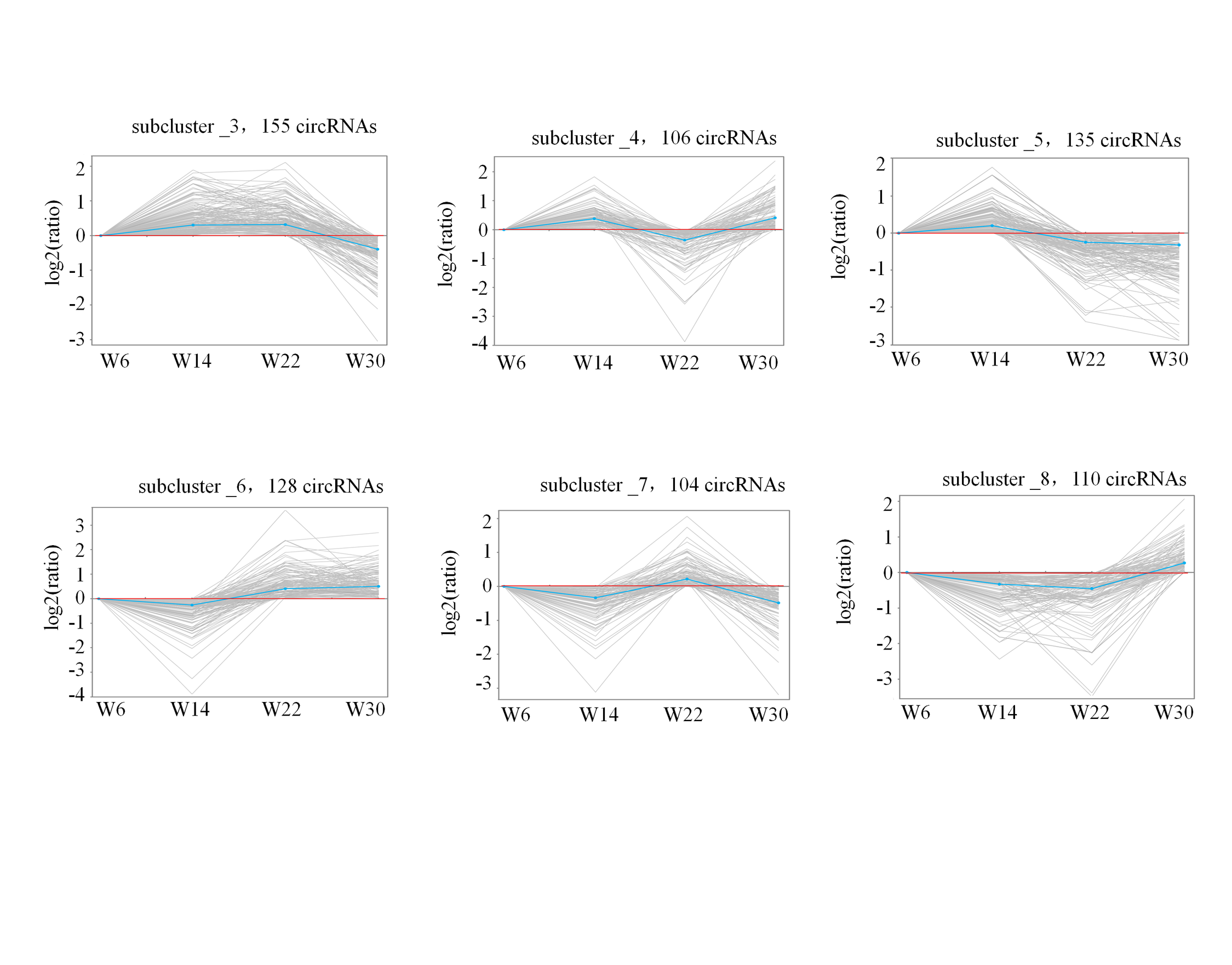

Supplement: S3 Fig — (TIF) [file pone.0249288.s003.tif]

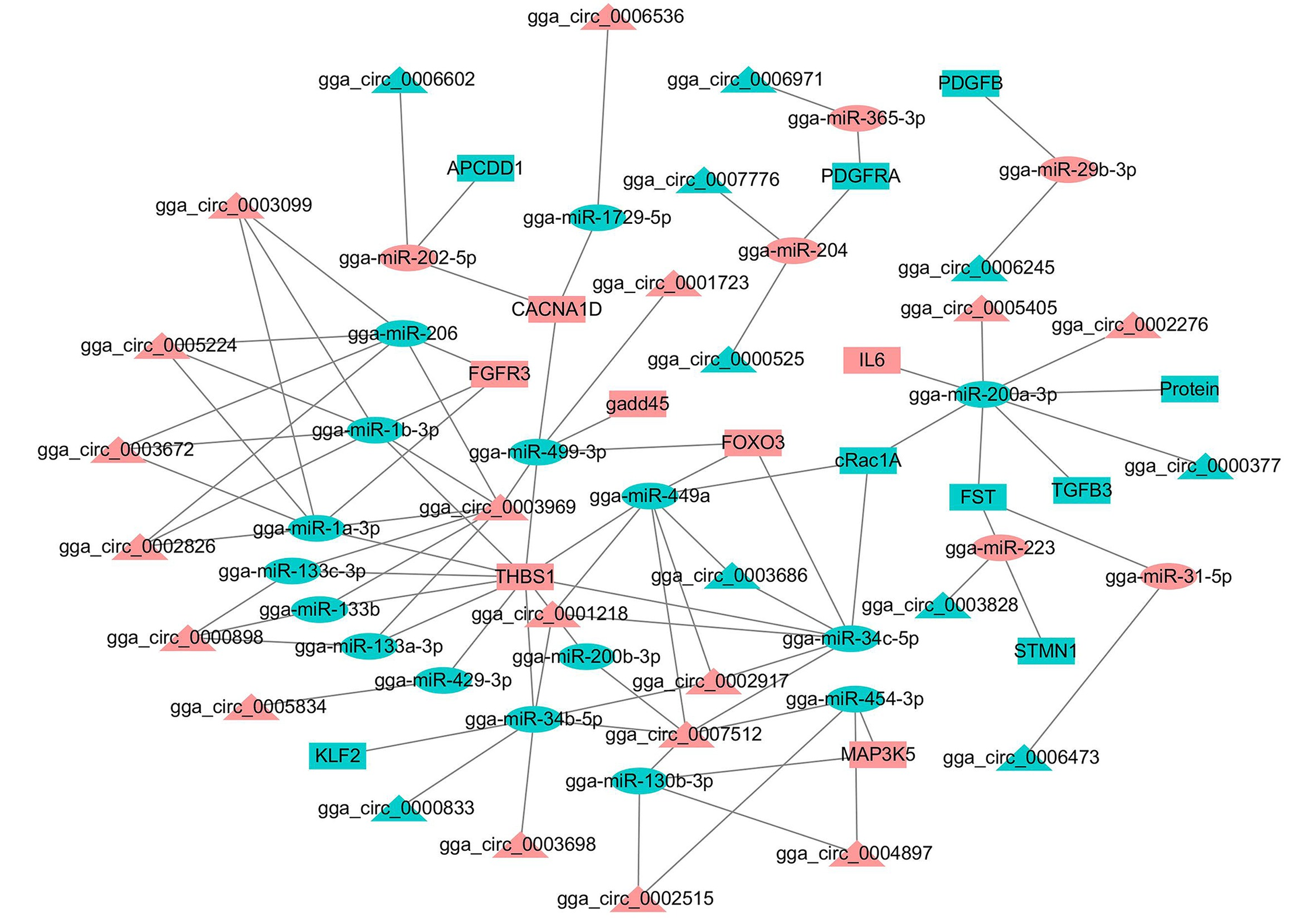

Supplement: S4 Fig — The ellipse, triangle and box nodes represent DE miRNAs, DE circRNAs, and DE mRNAs, respectively. Pink indicates upregulation, and blue indicates downregulation. The five pathways are the Wnt signaling pathway, FoxO signaling pathway, p53 signaling pathway, TGF-beta signaling pathway and MAPK signaling pathway. (TIF) [file pone.0249288.s004.tif]

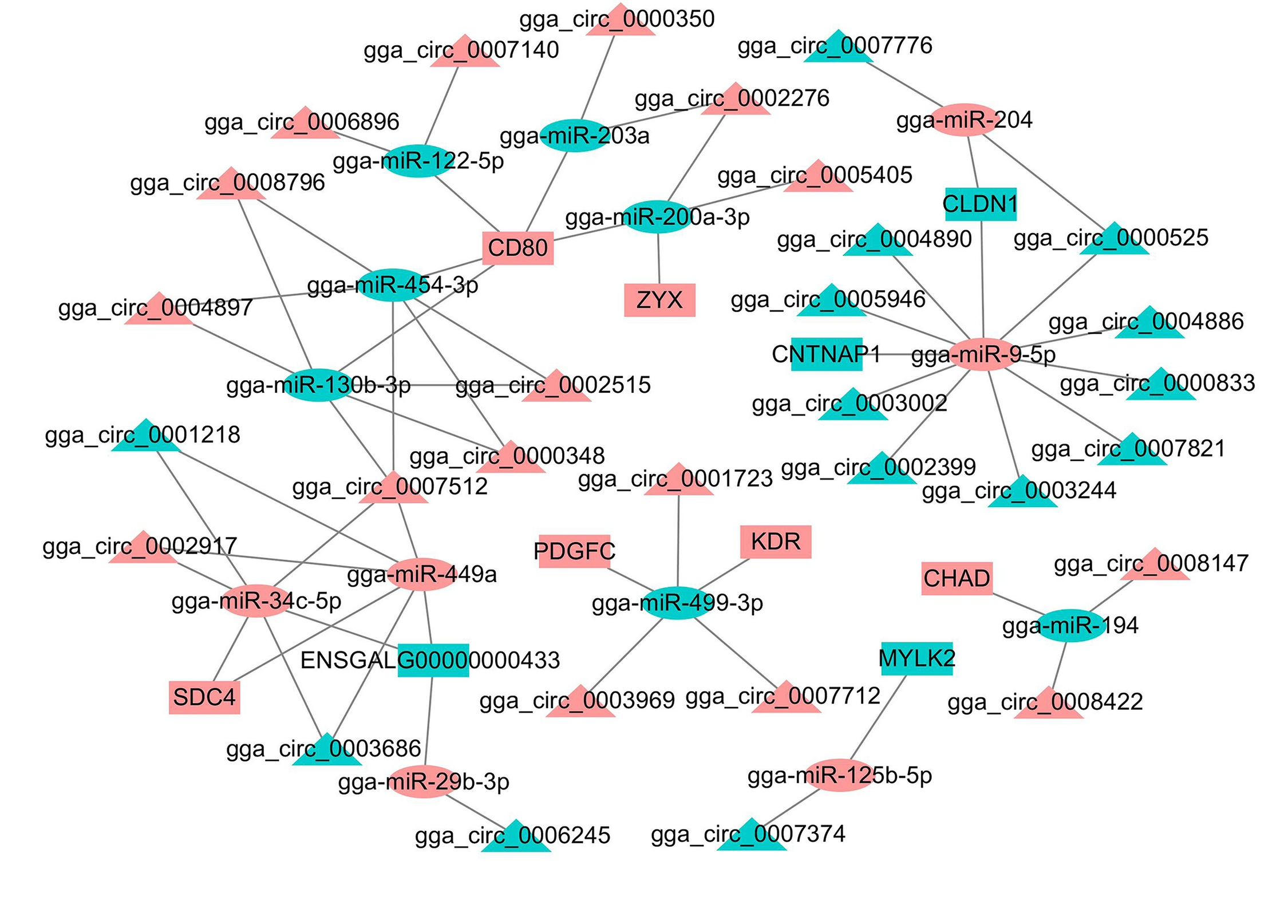

Supplement: S5 Fig — The ellipse, triangle and box nodes represent miRNAs, circRNAs, mRNAs, respectively. Pink indicates upregulation, and blue indicates downregulation. The five pathways are cell adhesion molecules, focal adhesion, adherens junctions, tight junctions and gap junctions. (TIF) [file pone.0249288.s005.tif]
