# Supplementary material for: Characteristics and expression profiles of circRNAs during abdominal adipose tissue development in Chinese Gushi chickens
Source: PLoS One. 2021 Apr 15;16(4):e0249288. doi: 10.1371/journal.pone.0249288 (PMC8049301; doi:10.1371/journal.pone.0249288)
Supplement: S1 Table — (DOCX) [file pone.0249288.s006.docx]

**S1 Table Primers used for the validation of circRNAs.**

| **circRNA primer name and type** | **Primer sequence (5′–3′)** | **Product length (bp)** |
| --- | --- | --- |
| gga_circ_0000348  divergent primer | F：TGCAAAGACTTCCGTATTGTCA | 195 |
|  | R：GTGGCATTGGGTCATCCGTA |  |
| gga_circ_0003969  divergent primer | F：ATGCACTTCCTCTTCGGAGC | 134 |
|  | R：CTGCGTGACTGTGACATGGA |  |
| gga_circ_0005065  divergent primer | F：AAGAAGTCCAGTGCACAGCC | 148 |
|  | R：ACAGTGCGGGTAAAAGCTGA |  |
| gga_circ_0001623  divergent primer | F：AGCTCCAGAATGCATCAGAGG | 189 |
|  | R：GCTTTTCCCAGAGTTCCGGT |  |
| gga_circ_0000833  divergent primer | F：CACGAGCCACAGTACGTGAT | 141 |
|  | R：GGAGCTATGAGCAGATGGGG |  |
| gga_circ_0004577  divergent primer | F：CCCATCCAAAGTTGTGGGGA | 189 |
|  | R：ACAGGGATTGCTAGGGAGGT |  |
| gga_circ_0003686  divergent primer | F：CATCCATCCCACATCCCTGG | 167 |
|  | R：CCAGCTTCAGTGAGCAAGGA |  |
| gga_circ_0003828  divergent primer | F：AGAAAAGGCCGGATGAAGGG | 174 |
|  | R：GCCAACCCCAAGGCTTCATA |  |
| gga_circ_0003244  divergent primer | F：AAGCCCAGAACATCACCGAG | 134 |
|  | R：ATTGGTTCCAGAGCAGGCTT |  |
| gga_circ_0002520  convergent primer | F:TGGCTTCACAAGCTTCTCAGAT  R: CCCACGGTGACCTATGATGG | 149 |
| gga_circ_0002520  divergent primer | F:ACCAAAGCCAGCCATCATAG  R:CTTGTGAAGCCAGTGTAGGTT | 100 |
