# Supplementary material for: Characteristics and expression profiles of circRNAs during abdominal adipose tissue development in Chinese Gushi chickens
Source: PLoS One. 2021 Apr 15;16(4):e0249288. doi: 10.1371/journal.pone.0249288 (PMC8049301; doi:10.1371/journal.pone.0249288)
Supplement: S1 Raw images — (PDF) [file pone.0249288.s010.pdf]

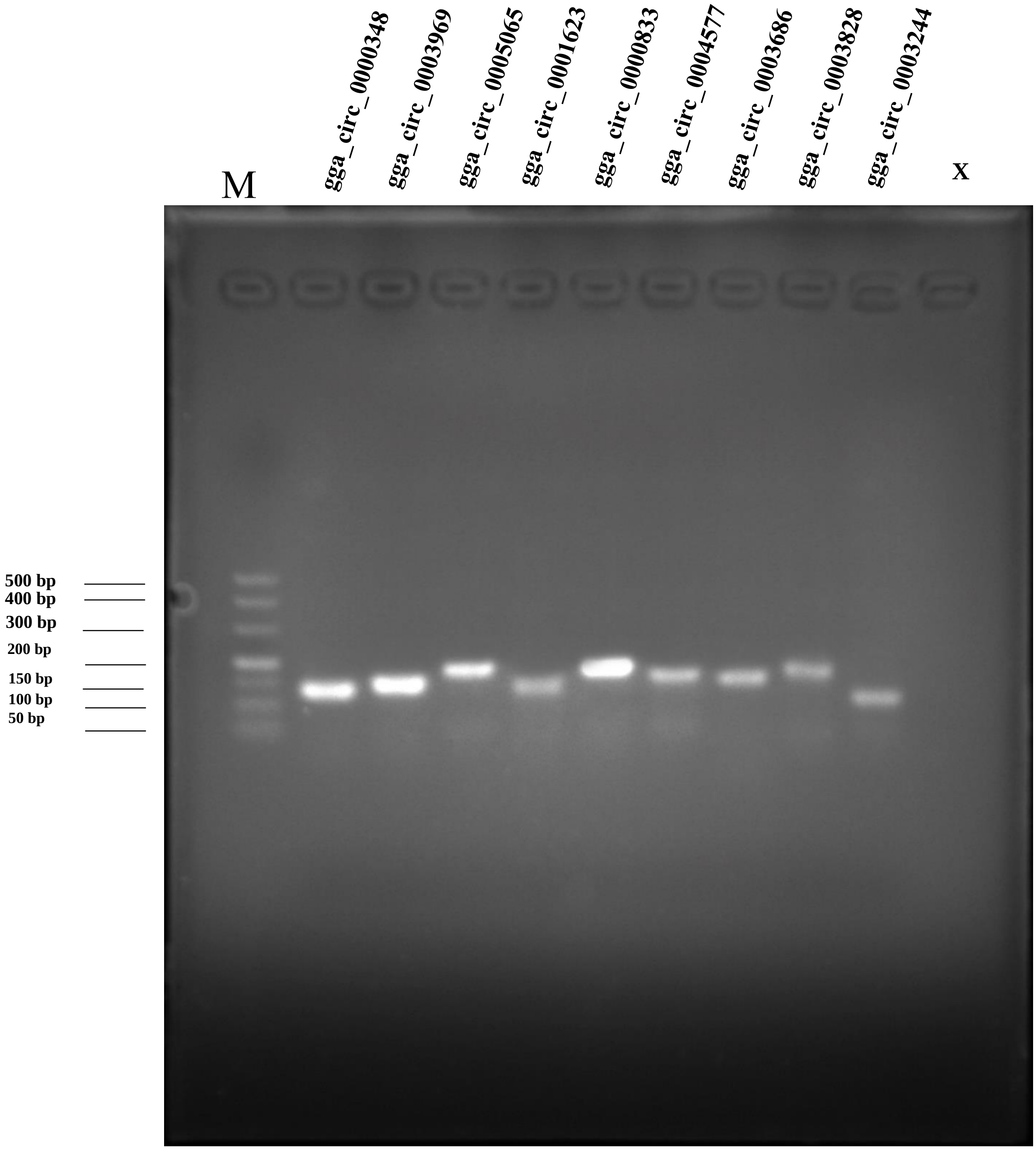

Note: This image is the original gel image of Figure 1A, taken by a gel imager. The sequence of experimental samples is shown in the figure. M represents DNA marker, X represents blank lane.

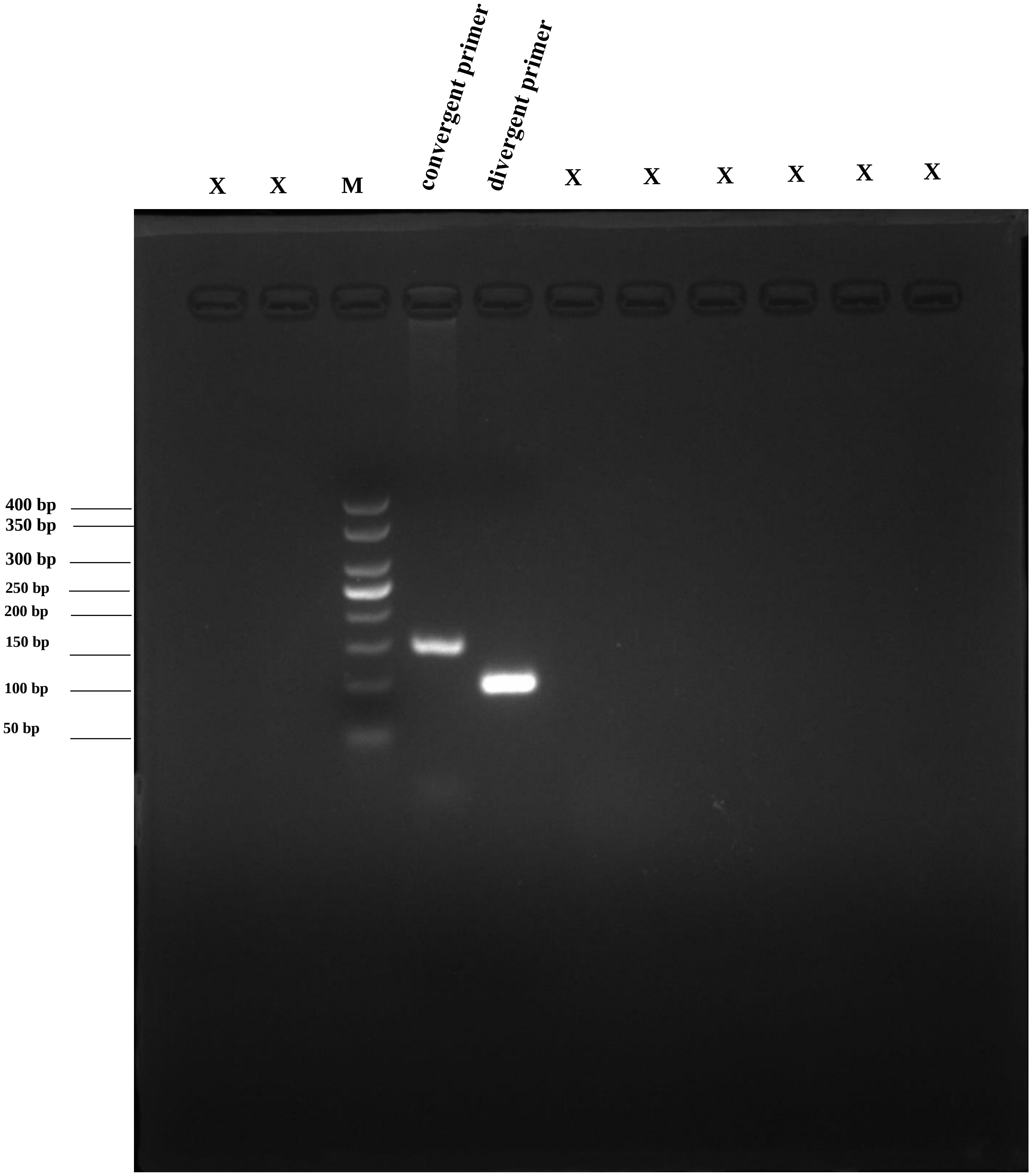

Note: This is the original gel image of Figure 9A, taken by a gel imager. The target bands were amplified by the convergent and divergent primer of gga_circ_0002520, respectively. M represents DNA marker, X represents blank lane.
